# The Mechanisms of Plastic Food-Packaging Monomers’ Migration into Food Matrix and the Implications on Human Health

**DOI:** 10.3390/foods12183364

**Published:** 2023-09-07

**Authors:** Celia Muzeza, Veronica Ngole-Jeme, Titus Alfred Makudali Msagati

**Affiliations:** 1 Institute for Nanotechnology and Water Sustainability (iNanoWS), College of Science, Engineering and Technology, University of South Africa, Science Campus, Roodepoort, Johannesburg 1709, South Africa; 2 Department of Environmental Science, College of Agriculture and Environmental Sciences, University of South Africa, Science Campus, Roodepoort, Johannesburg 1709, South Africa; ngolevm@unisa.ac.za

**Keywords:** food-packaging material (FPM), monomer migration, endocrine-disrupting compounds (EDCs)

## Abstract

The development of packaging technology has become a crucial part of the food industry in today’s modern societies, which are characterized by technological advancements, industrialization, densely populated cities, and scientific advancements that have increased food production over the past 50 years despite the lack of agricultural land. Various types of food-packaging materials are utilized, with plastic being the most versatile. However, there are certain concerns with regards to the usage of plastic packaging because of unreacted monomers’ potential migration from the polymer packaging to the food. The magnitude of monomer migration depends on numerous aspects, including the monomer chemistry, type of plastic packaging, physical–chemical parameters such as the temperature and pH, and food chemistry. The major concern for the presence of packaging monomers in food is that some monomers are endocrine-disrupting compounds (EDCs) with a capability to interfere with the functioning of vital hormonal systems in the human body. For this reason, different countries have resolved to enforce guidelines and regulations for packaging monomers in food. Additionally, many countries have introduced migration testing procedures and safe limits for packaging monomer migration into food. However, to date, several research studies have reported levels of monomer migration above the set migration limits due to leaching from the food-packaging materials into the food. This raises concerns regarding possible health effects on consumers. This paper provides a critical review on plastic food-contact materials’ monomer migration, including that from biodegradable plastic packaging, the monomer migration mechanisms, the monomer migration chemistry, the key factors that affect the migration process, and the associated potential EDC human health risks linked to monomers’ presence in food. The aim is to contribute to the existing knowledge and understanding of plastic food-packaging monomer migration.

## 1. Introduction

In the past century, humans have mainly been sustained by locally grown seasonal foods that could meet their food demand within the food shelf life [1]. However, with the current technological advancements coupled with high levels of industrialization and better standards of living, which have catalysed the formation of large cities that are highly populated, there is virtually no space to grow food crop [1]. The scientific advancements in the last fifty years have nevertheless contributed to an increase in and variety in world food production [2], but demand remains high! Globally, these and other factors have resulted in the need to transport and store a variety of foods over long distances to consumers and for long times, respectively, taking a longer time beyond the storage life of the foods as a result. Due to the fact that the chemicals within food may be subjected to various environmental conditions, including oxygen (O_2_), water vapour (H_2_O), and light, during transportation and storage, which possibly leads to microbial contamination or a loss of valuable properties, such as nutrition, colour, texture, and edibility [3], modern society households mainly depend on food refrigeration for various plastic food-packaging types as a method of food preservation. Industries also rely on numerous modern preservation techniques (high-pressure technology, irradiation, and hurdle technology) [3,4] and traditional preservation techniques, including packaging to meet the current demands of economic preservation and keeping food stable and safe to maintain the food quality [5] for consumer satisfaction. Therefore, in the food industry, food packaging serves as an indispensable multifunctional element and a sector currently representing a dynamic part of most economies, which currently contributes significantly to the Gross National Domestic Product (GDP) [6,7,8,9]. Research reports further speculate that, due to population expansion, the world’s food supply will need to expand by 50% by 2050, which will consequently trigger a significant demand for food-contact materials [10,11]. In the developed world, the key driving factors, such as the increased plastic recycling infrastructure, the global population growth, a rise in feeding with processed and take-away foods due to consumers’ busy lifestyles, and numerous other factors, increase the demand for convenience foods, which contributes positively towards the growth of the food industry [9]. In countries with emerging economies, South Africa, for instance, is a giant polymer producer of different food-packaging types. For the projected period of 2017–2027, the South African packaging market is anticipated to convert to an extraordinary growth region with a compound annual growth rate (CAGR) of 7.8% [12]. Plastic consumption per capita is particularly being projected to increase due to urbanization, urban–rural migration, and an increase in middle-income households [13]. As in the rest of the world, their modern food packaging makes use of ceramics, glass, metal, paper, paperboard, wood, and plastic material types to package a variety of retail and domestic food products [14,15,16,17]. Plastic dominates the packaging industry because it has numerous food-packaging advantages compared to its disadvantages, which have enormously contributed to its preference as a food-contact material to package various food items (Table 1). As such, it accounts for about 52% of most local markets [18]. Polypropylene (PP), polyethylene (PE), polyvinyl chloride (PVC), polystyrene (PS), polyethylene terephthalate (PET), and polyurethane (PU) are the polymer types globally dominating the plastic food-packaging industry [15,19,20]. The word ‘plastic’ is normally utilized to describe materials synthesized through the addition, condensation, or cross-linking polymerization of monomer units [21,22]. Plastics are present in various rigid and flexible forms to which, in addition to monomers, additives, for instance, plasticizers, adhesives, coatings and solvents, antioxidants, thermal and light protectants, and graphic information, are added [23] to ensure the characteristics needed for their function [16,24,25]. Heat and pressure is then applied to mould the polymers and to obtain the required final products and shapes, such as films, trays, and bottles [26]. 

However, despite the significant different roles that plastic food-packaging materials fulfil both domestically and within the food industry sector, in their natural state, plastic food-contact materials are limited in their ability to provide the required mechanical and barrier properties. This is because, without additives, for instance, plastic food packaging is limited in its food-packaging role. Additionally, most importantly, the scientific research evidence reveals that the chemical substances utilized during polymer synthesis, including the main building blocks (monomers), leach throughout the plastic product’s life cycle [28,29,30]. More concerning is that, compared to other packaging material types, research confirms that chemical migration is more likely to occur in plastic packaging [31]. Migration describes the mass transfer of chemical substances from a higher-concentration region (the food-contact side) to a lower-concentration region (usually the food) until equilibrium is reached [32]. Monomers have been implicated as endocrine-disrupting compounds (EDCs) linked to serious human health problems that compromise consumer health, especially the safety of pregnant mothers and children, who make up the vulnerable group in the population [11,33,34,35,36,37,38,39,40,41,42,43,44]. Up to 906 chemicals have been linked with plastic food packaging of which 63 are classified as human health hazards; 68 are classified as environmental hazards; 7 are classified as persistent, bioaccumulative, and toxic; 15 are classified as endocrine-disrupting compounds (EDCs); and 34 are classified as potential EDCs [23]. However, these known values of the different chemical categories in plastic food packaging are but an insignificant proportion given that about 10,000 chemicals show potential capabilities of migration from plastics into food when subjected to various physicochemical conditions [23,32,45,46] during processing, transport, storage, and food preparation. However, to date, more than 2000 substances lack toxicological and detailed descriptions of their scope of use [23] due to numerous reasons, including the prevailing limitations in structure elucidation as a result of the lengthy modern analytical procedures used for the detection of monomers [47]. Additionally, the research contributions, which are mostly from the developed world, are biased on a few commonly known hazardous substances occurring in plastic food packaging, such as Bisphenol A and styrene (monomers) and phthalates (plasticizer additives) [48]. This is primarily due to the fact that it is difficult to find comprehensive information on these chemicals, including plastic monomers, in the public [20,28]. It is important that all the chemicals, including monomers, in food packaging are well accounted for so that their potential harm to humans is well understood and reduced. This is especially true because consumers interact with plastic packaging daily. The low amount of accounted data hence suggests that humans are susceptible to unknown harmful food-packaging chemicals daily. Additionally, there is scanty information on the treatability and level of the human health and environmental toxicity of the numerous known and unknown plastic food-contact chemicals, including monomers. Due to the fact that most of these substances have hardly been studied, about 1327 substances are, as a result, insufficiently governed across the world. Consequently, 901 substances are accepted for utilization in plastic food-packaging materials [49], but they have unknown impacts on human health. There is, therefore, an outcry for widespread research to ensure that food packaging, especially plastic packaging, maintains its main role of protecting food. A sustainable circular plastic economy which reduces and, even better, prevents the use of hazardous chemicals as well as increases information accessibility is therefore essential. Although research studies on plastic food-packaging chemical compounds’ migration into food products are widely reported [24,26,50,51,52], previous review works mostly focus on the migration of additives, challenges in additive analyses in the food and biological matrices [53], and impacts on human health [54,55]. To add to the literature, this work provides a critical review on monomer migration, including the monomer migration mechanisms and chemistry in versatile plastic food packaging. Furthermore, monomer endocrine-disrupting effects are currently speculated to be one of the major reasons for most of the current global chronic illnesses. The aim is to shift the focus of the relevant authorities, especially in emerging-economy countries, from only addressing the environmental pollution of plastic packaging to also urgently addressing and regulating its health impacts due to migrating monomers. This is because existing plastic packaging legislative authority regulations, for instance, those in South Africa, are biased towards environmental pollution. This is evidenced by the numerous legislative authority regulations, such as the National Environmental Management Waste Act, Act 59 of 2018, and the National Water Amendment Act, Act 27 of 2014 [56,57], and yet policies, education, and awareness that address the human health plastic toxicity effects are lacking!

## 2. Food-Contact Chemicals in Plastic Food-Packaging Types

Plastic food-packaging materials (FPMs) comprise different contact chemicals and a variety of synthetic materials made from different chemical compounds and their combinations thereof (Table 2) and are used to keep food safe during the transportation of diverse food products [58]. Table 2, for instance, illustrates the composition of a plastic yoghurt container.

A detailed analysis of the food-contact chemicals (FCCs) worldwide reveals that there are about 12,285 intentionally added substances (IASs) [63], some of which are the building blocks (monomers) of plastic packaging materials. Much more, although difficult to predict, there are nonintentionally added substances (NIASs) from numerous other possible reactions and transformations [23]. Additionally, there are various contaminants from the recycling processes in the synthesis of food-contact materials [64]. However, the global challenges related to food safety suggest that the current scientific knowledge demonstrates a limited detailed understanding of all the possible materials in a packaging type. This is more than important especially because, during the last few decades, plastic food-packaging materials have transformed significantly, with new materials, designs, and technologies such as microwaveability, evolving to enable packaging to respond to the increased demands of the modern consumer lifestyles [35]. As such, the current standards of manufacturing compliance may not sufficiently account for the possible migration implications of the packaging material. Table 3 and Table 4 show the monomers and some additives added to common plastic food-packaging types. Interesting to note is that some additives, Bisphenol A, for instance, are also monomers in some plastic food-contact material types.

Additives are added to a polymer for various functions (Table 4), such as to improve the overall characteristics of the polymer in accordance with its suitability for its end use [20]. However, they bind reversibly to the polymer system, and, as a result, monomers also easily leach into the food [20,70]. 

**Table 4:** Some additives present in plastic food-packaging materials.

Additive Name	Function	Structure	Reference
Plasticizers	Increase the workability and flexibility of final product	Bisphenol A (BPA) 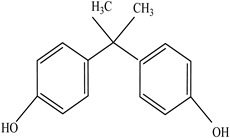 Phthalates 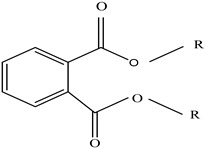	[58,71]
Antioxidants	Scavenge free radicals, reducing the oxidation process that exposure to light causes in polymers	Butylated hydroxytoluene (BHT) 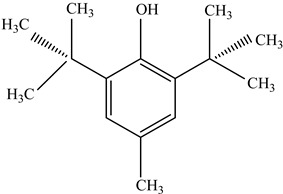 Irganox 1010 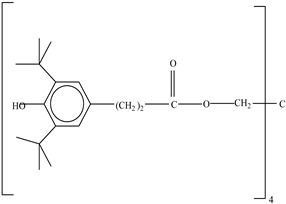 Bisphenol A (BPA) 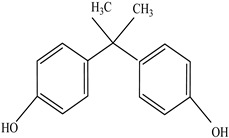 Butylated hydroxyanisole (BHA) 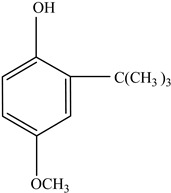 Ionox 100 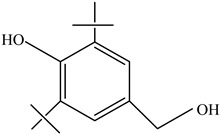 Irganox 1076 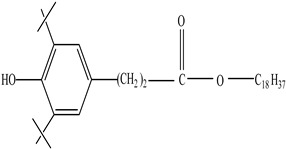	[72]
UV protectants	Stabilize polymers and prevent degradation	UV-326 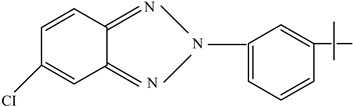 UV-234 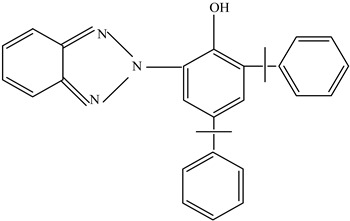 UV-P 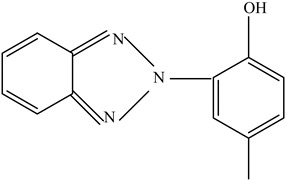	[73]

Recently, due to environmental concerns as a result of the non-biodegradability of plastics [74,75], there has been a drive towards the development of nontoxic eco-friendly biodegradable plastics [74]. Generally, three types currently exist based on their source of origin and method of production amongst which are various biopolymers produced through the chemical synthesis of renewable bio based monomers (Table 5). 

However, despite the seemingly acceptable organoleptic, mechanical, and chemical properties of biodegradable food packaging [77], its commercial application to date has been limited for numerous reasons, including the non-systematic knowledge on the migration of chemicals, including monomers [78]. This is because the utilized monomers have a low average molecular weight with the potential to diffuse through the polymeric matrix when utilized in food packaging. Strategies to improve biodegradable packaging performance involve the addition of a variety of substances, such as nano fillers and plasticizers, which also adds to the concerns of their migration into the food [79,80,81,82] The increased addition of additives leads to undesirable interactions and the consequent migration of substances that may be more or less relevant for one than for the other [79]. Furthermore, the current research is centred on food simulants rather than real food products [78] and is concentrated on a few biopolymers, such as PLA [78]. The toxicological effects on animals are also lacking. Moreover, most countries of emerging economies do not have nanoparticle (NP)-specific regulations for aquatic systems, including wastewater treatment plants, where egested food with NP contaminants is finally deposited, which presents accurate, scientifically proven, and confirmed detection difficulties for the safe migration limit in food-packaging films in the respective countries [83]. Conclusively, more research on the migration of both components is essential to draw results on the safe utilization of biodegradable packaging with regards to chemical migration. 

## 3. Packaging Monomers as Sources of Endocrine-Disrupting Compounds (EDCs) in Foods

The human body comprises tissues that interact with each other by means of hormones that control, for instance, reproduction, the early developmental processes, and the tissue and organ functions throughout adulthood [84]. Exogenous (non-natural) substances known as endocrine-disrupting compounds (EDCs) imitate the effects of natural hormones, preventing their production, release, transport, metabolism, binding, and elimination, which are essential for maintaining homeostasis, reproduction, and the developmental and behavioural processes in the human body, consequently causing adverse health effects [84]. Endocrine-disrupting compounds, such as monomers, in plastic food packaging thus disrupt the coordination and, consequently, the efficient functioning of the endocrine system, which is responsible for the regulation of various body processes [85,86,87,88]. Monomers are amongst the hundreds of EDCs globally utilized within the plastic food-packaging industry [89], such as additives (plasticizers, oxidants, and preservatives). Their EDC effect presents adverse human health problems across all consumers, with more health impacts on the younger generation, which poses a great challenge for future generations. Information on the types of EDCs present in the various plastic packaging materials is presented in Table 6.

## 4. Monomer Migration into Food in Food Packaging

**Figure 1:**
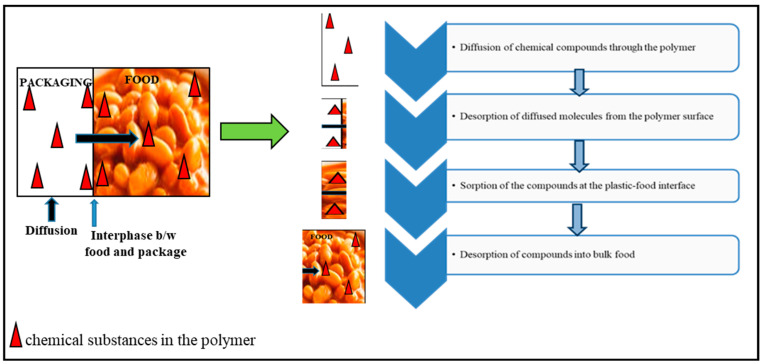
Monomer migration process.

Since plastic packaging is produced through a polymerization process where monomers or building blocks are linked together, monomer residues are always present in plastics, although they are present at generally low concentrations of 0–2%. This is because not all added reactants will complete the reaction [30]. Furthermore, the majority of low-molecular-weight substances, like LDPE, are not covalently attached to the polymer chain. Instead, they take the form of branching chain structures, which prohibit the monomer units in the polymer chain structure from being packed closely together. As a result, these and residual monomers are able to diffuse all over the polymer matrix [32]. However, although monomers are generally stable and nontoxic when bound by the polymer matrix, interactions with food make them harmful and cause them to affect human health when they are later consumed with the food, and their concentrations increase in the body [39,99]. The migration process, which is influenced by numerous parameters, is divided into the following four primary steps. These are chemical compound diffusion through the polymers, diffused molecule desorption from the polymer surface, compound sorption at the plastic–food interface, and compound desorption throughout the food [20] (Figure 1).

Due to the fact that migration may introduce unwanted and dangerous chemical substances, which deteriorate the nutritional value, safety, and organoleptic qualities of packaged foods, the process is thus undesirable. Nonetheless, some transfer is unavoidable because food must be packaged before being purchased by the consumer [100]. To date, evidence from numerous researchers reveals monomer migration results from polystyrene, polyamides, polycarbonates, polyvinyl chloride, and polyurethanes plastic types into different foods and food simulants [101]. However, food simulants are commonly utilized in laboratory investigations to counteract the complexity of the physical structure of food, to understand migration more clearly, and for regulatory compliance reasons [102]. Commonly utilized food simulants include water for aqueous environments, 3% acetic acid for acidic food simulants, 10% ethanol for alcoholic food simulants, and refined olive oil for fatty food simulants as guided by the European Union [103] for migration tests on plastic food-contact materials. To verify the overall safety of the plastic contact material, the European Food Safety Authority (EFSA), for instance, indicates that the maximum limit of overall migration (the total of all the substances together) into a packaged food sample should be 60 mg kg^−1^ of the food or 10 mg dm^−2^ of the packaging material [20]. However, there are various complications with migration tests, including that the tests have long experimental workflows. Additionally, based on different cultures, which translate to how food is prepared in different nations, perhaps accurate and reliable research on monomer migration should focus on using real foods rather than food simulants to incorporate the different food preparation methods and spices that could otherwise affect chemical migration. The resultant OM and SM is, therefore, likely to be complicated and not comprehensively explained by food simulants. As such, simulants in comparison to real food samples risk omitting other possible interactions between food and the food’s packaging. Furthermore, some materials, for instance, absorbent packaging materials, pose problems for accurate OM and SM testing. Migration tests also need to determine the temperature and humidity conditions to imitate the stress generation conditions that facilitate investigating the behaviour of the packaging material [32], which poses a huge challenge in the evaluation of OM for different food-packaging materials and, therefore, in that of the possible health and environmental effects.

### 4.1. Migration Mechanism Processes in the Migration of Monomers into Food

Migration occurs in a number of different ways, including contact, penetration, gas-phase, condensation, and set-off migration [104] 

***Contact migration***

**Figure 2:**
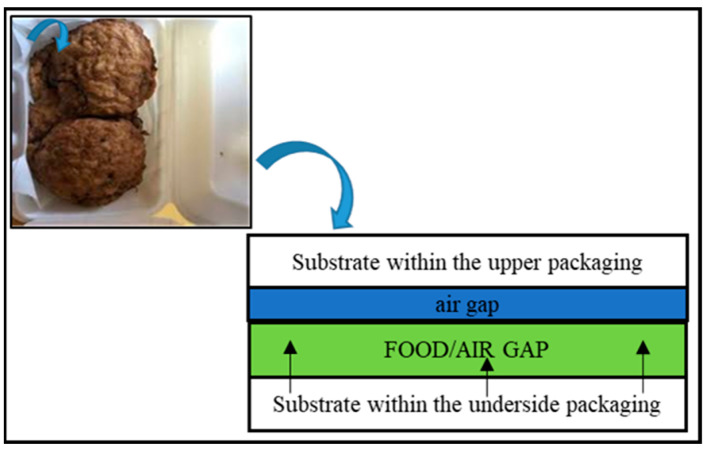
Contact migration mechanism in a pizza box.

A direct substance transfer from the packing material’s food-contact surface into the packed food is referred to as contact migration. Examples of contact transfers include the migration of materials from a plastic tub tray or wrapping into food and from a cardboard pizza box to the underside of the pizza (Figure 2) [104].

***Condensation/distillation migration***

**Figure 3:**
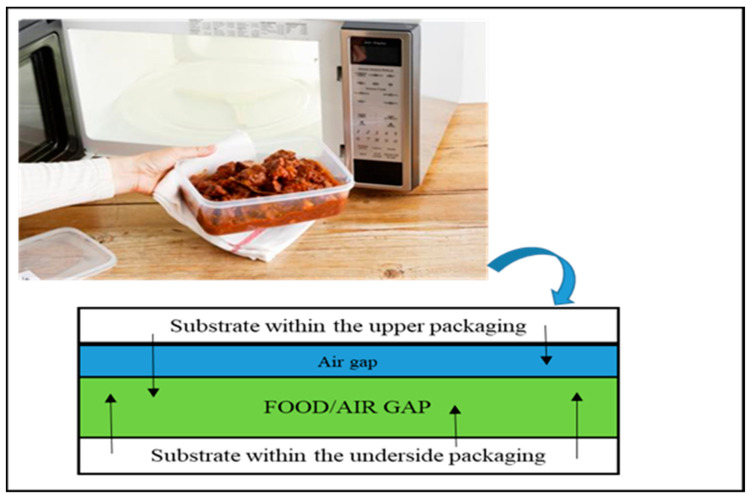
Condensation/distillation mechanism in microwave heating.

Condensation migration involves the leaching of chemical substances, particularly volatile components from food-packaging materials to food during heating stages, such as sterilization or boiling [104]. However, several migration studies’ findings reveal distillation migration even before the above heating stages. As such, condensation leaching examples include microwave heating to cooking in cartons, trays, or plastic food containers (Figure 3) [104]. 

***Gas-phase migration***

**Figure 4:**
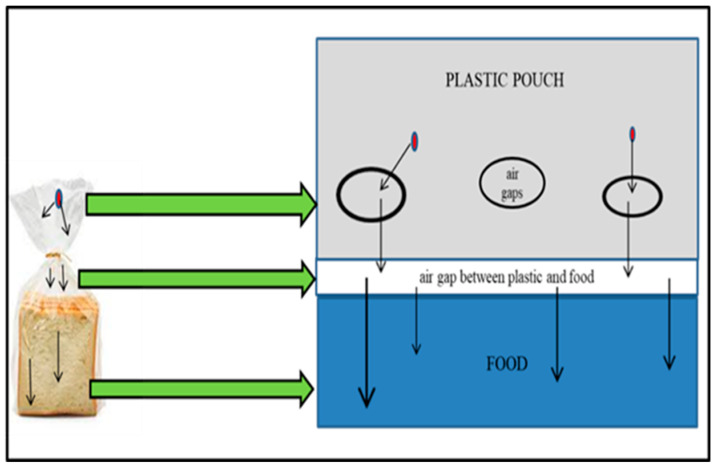
Gas-phase migration mechanism in plastic bread packaging.

Gas-phase migration relates to the permeation of volatile chemicals from the packaging coating on the outer layer through the airspaces within the plastic packaging and between the packaging material and food into the food through diffusion (Figure 4). Examples include the diffusion of mineral oil into meals through a plastic inner pouch (depending on the material’s barrier qualities), an airspace within paper packaging, or a second airspace between the packaging and food [105]. 

***Penetration migration***

**Figure 5:**
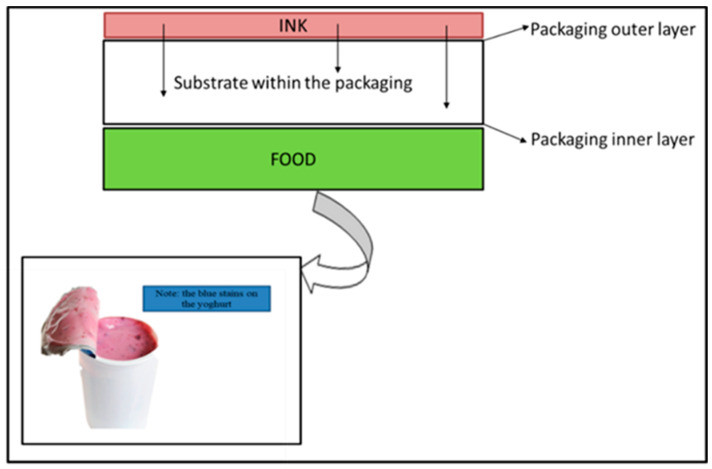
Penetration migration mechanism in a yoghurt container.

The penetration type of migration is the diffusion of chemical substances from the packaging non-food-contact surface (often a coated or printed surface) through the substrate and onto the packaging’s food-contact surface (the inner layer) (Figure 5). Once the migrating chemicals are on the food-contact surface, they then leach into the food through gas-phase or contact migration, contaminating the food [104]. 

***Set-off migration***

**Figure 6:**
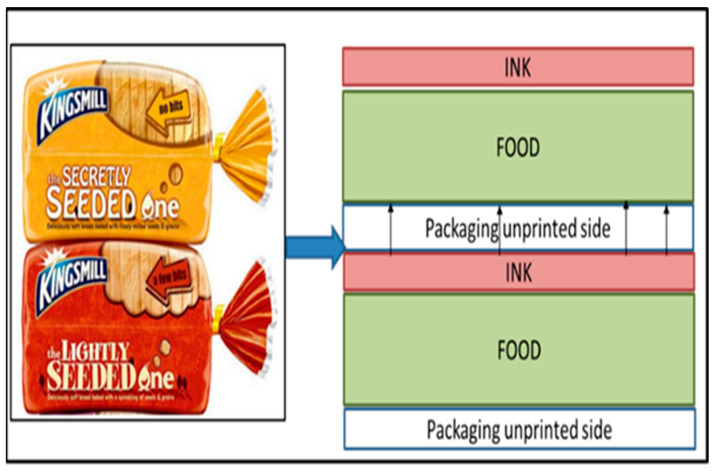
Set-off migration mechanism in stacked bread.

Set-off migration describes chemical substance diffusion from the coatings, varnishes, or printed ink present on the outer printed non-food-contact side of the package material through the substrate towards the inner food-contact side due to the stacking of the printed items (Figure 6). 

The set-off kind of migration can be either obvious or invisible. Once chemical compounds are on the food-contact surface, they are subsequently transferred throughout the food through gas-phase or contact migration, contaminating the food [104]. 

**Figure 7:**
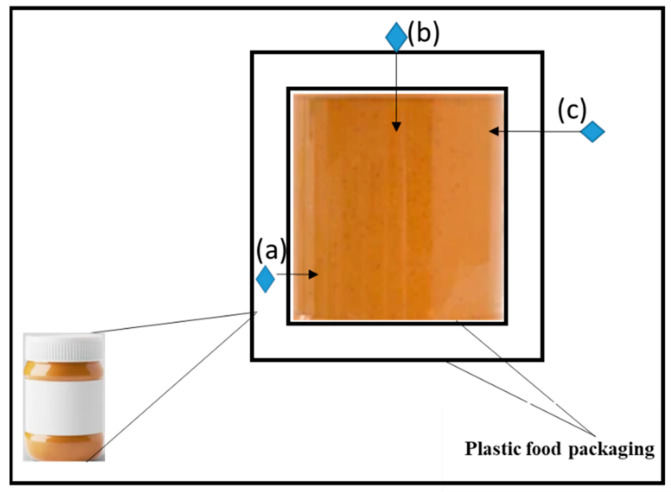
Illustration of the migration mechanism in plastic food packaging.

Due to the various chemicals present in plastic food packaging, small molecules, including monomer residues, oligomers, additives, reaction by-products, and adhesive components (a) as well as printing inks (b) from the outer layer of the packaging or from others in a stacked pile, diffuse and leach from the plastic material into the food (Figure 7) [62]. 

Several studies agree that, through the influence of several factors, migration either follows a set-off, contact, gas-phase, or penetration migration mechanism process depending on the present situation [105], and they further illustrate that in recycled plastic food packaging, environmental toxins, like pesticides, detergents, and persistent organic pollutants (c), are absorbed into the plastic packaging and are subsequently released again.

Some of the identified leaching monomers from plastic food-packaging materials that are particularly labelled as problematic include Bisphenol A, styrene, Bisphenol A diglyceride ether (BADGE), and caprolactam.

**Bisphenol A (BPA)** added as an antioxidant to polymers, for instance, can potentially migrate from PC or plastic resins commonly used in cans [106]. Its migration into different foods, including water and 10% and 50% ethanol, in PC and various plastic containers, such as PC baby bottles, baby bottle liners, non-PC baby bottles, and recyclable PC drinking bottles, has been recorded from the environment and from the can linings and PC bottles through investigations of several factors [107], and BPA’s migration in evaporated milk, carrots in brine, minced beef in gravy, spring vegetable soup, and a food simulant (10% ethanol) has also been studied. The amount of migrated BPA was significantly higher in 10% ethanol (68.3 ± 9.0 µg kg^−1^) compared to the following foods: minced beef (53.8 ± 7.6 µg kg^−1^), milk (49.8 ± 10.9 µg kg^−1^), carrots (47.2 ± 5.1 µg kg^−1^), and soup (45.7 ± 5.0 µg kg^−1^). Bisphenol A diglyceride ether (BADGE) is also an epoxy resin polymer monomer utilized in internal food can linings. In a separate study [108], the amount of BADGE and BPA that leached into distilled water from two different can types that packaged tuna fish and jalapeño peppers was examined. The conclusions based on the study results are that both monomers migrate, although there are different factors influencing their overall migration.

**Styrene monomers** are always present in PS, acrylonitrile-butadiene-styrene, and polyamide packaging materials [109], which are widely utilized to package a range of dairy products, such as ice cream and yoghurt; bakery products; juices; meat; and fresh produce [110]. However, research reveals that residual styrene monomer levels vary in similar packaging materials utilized to package similar products within different countries (Table 7) [111]. Furthermore, migration studies reveal that styrene monomer migration is dependent on several factors. In [112], studies on styrene migration from various PS food-contact packaging materials, including egg cartons, meat trays, plates, and cups, into oil showed that migration increased within days. With the exception of drink cups, migration was also proportional to the square root of the time increase. In a separate study with hot drinks, the migration of styrene strongly depended on the temperature and amount of fat in the hot drinks [113]. The styrene monomer migration level results in µg/L varied from 0.61 to 8.15 for hot tea, 0.65 to 8.30 for hot milk, and 0.71 to 8.65 for hot cocoa milk in GPPS (general-purpose polystyrene) cups and from 0.48 to 6.85 for hot tea, 0.61 to 7.65 for hot milk, and 0.72 to 7.78 for hot cocoa milk in HIPS (high-performance polystyrene) cups at different temperatures and times [114]. The findings showed that hot cocoa milk had the highest degree of styrene leaching [114]. Further studies on styrene migration in aqueous and oily foods also revealed less styrene migration because the monomer is hydrophobic [115]. However, a recent study reveals that the effect of the fat content on the migration of styrene is insignificant in relation to the variability of other parameters [111]. 

Ref. [116] studies on caprolactam monomer migration from nylon 6 and nylon 6/66 polymers to oil when cooked in an oven showed that the nylon 6/66 oligomers that migrated due to the above made up nearly 43% of the existing oligomers in the utilized packaging material. In addition, Ref. [117] investigated how caprolactam moved from nylon 6 packaging to 95% ethanol. The samples analysed also included poultry breasts, ham, pate, turkey blanquettes, and bologna sausages. The findings showed that the migration of caprolactam was above the set EU standard of 15 mg kg^−1^ in 35% of the packaging for bologna sausage, 33% of the turkey blanquette packaging, 100% of the pate packaging, and 100% of the packaging for poultry breast [118]. Based on the continuous evidence of monomer leaching from plastic food packaging into the food, it is therefore important that, globally, industrial policies speak and implement enforcement measures that will ensure the compulsory synthesis of plastic packaging materials with efficient polymerization processes. 

The concept of pyrolysis, which involves the thermal breakdown of organic molecules at a moderate temperature and in the absence of oxygen [119], can be used to inform on the bond dissociation energies (BDEs) of the monomers of different plastic polymers. The bond dissociation energy is a crucial thermodynamic quantity that represents the minimum energy required to break chemical bonds, in this instance, the monomer bonds from the polymer structure, so that they leach into the food. In addition, it also exemplifies the chemical activities of the free-radical reactions [120] in the plastic polymers, which are important in the chemical migration phenomenon and, thereafter, in food safety and quality. The larger the BDE, the stronger the chemical bond is, and the less likely the bond is to break. The bond dissociation energies of four common plastic packaging polymers calculated using two-density functional theory methods (DFTs) (B3P86/6 with the −31 G (d,p) basis set and M062X/6 with the −31 G (d) and −31++G (d,p) basis sets) are shown in Table 8. 

The BDE findings show that the main chain C-C bonds for PP (329.5) and PS (291.7) are generally weak. A comparison of the four polymers therefore suggests that the thermal stabilities of the four polymers are in the order of PE > PP > PS > PVC. Based on the bond dissociation energy equation indicated in Equation (1), the bond association constants of the monomers of the respective polymers calculated using Equation (2) are shown in Table 9 (coloured):

(1) $$\left[P\right]+\left[L\right]\;\;\begin{array}{c} K_{a}\\ ⇌\;\;\\ K_{D} \end{array}\;\left[PL\right]$$

where [P] is the protein concentration/polymer, [L] is the ligand concentration/monomer/any molecule that the polymer binds, K_a_ is the association constant, K_D_ is the dissociation constant, and [PL] is the concentration of the protein ligand complex.

(2) $$K_{a}=\frac{1}{K_{d}}$$

### 4.2. Migration Mechanisms Involving Different Chemistries of Monomers

Throughout the previous twenty years, scientific research studies demonstrated that leaching from packaging materials into food simulants and food is a predictable diffusion process within the polymer network [123]. However, the migration chemistry mechanisms for different migrants, including monomers, are not the same. This is because, according to [124], simulating the migration process of each migrant from, for example, plastic packaging materials to food is difficult. The migration mechanism chemistries of two commonly researched monomers, for instance, are shown below: 

***Bisphenol A***

**Figure 8:**
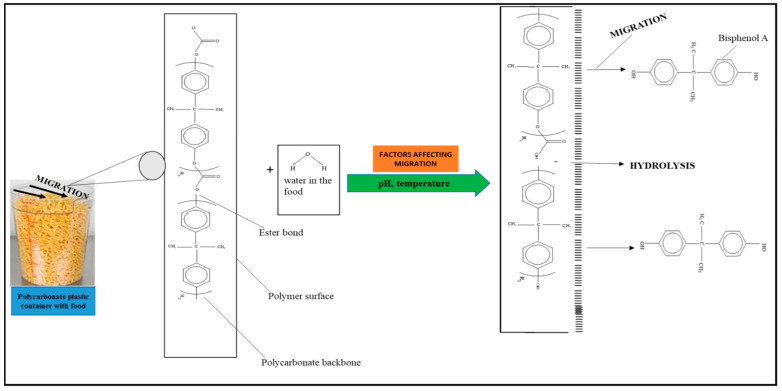
Hydrolysis migration mechanism of Bisphenol A from polycarbonate.

Two different processes explain the leaching of Bisphenol A from polycarbonate polymeric materials. These processes are pH-dependent hydrolysis/decomposition, which occurs over time at the polymer surface (Figure 8), and the diffusion-controlled release of the leftover BPA monomers from the polymer [125].

However, a comparison of the two migration methods shows that hydrolysis at the surface is the primary source of BPA migration into aqueous media and that it occurs at the carbonate–ester linkages of the PC backbone. In contrast, diffusion-controlled release only contributes to a relatively small part in overall migration [125].

***Styrene***

**Figure 9:**
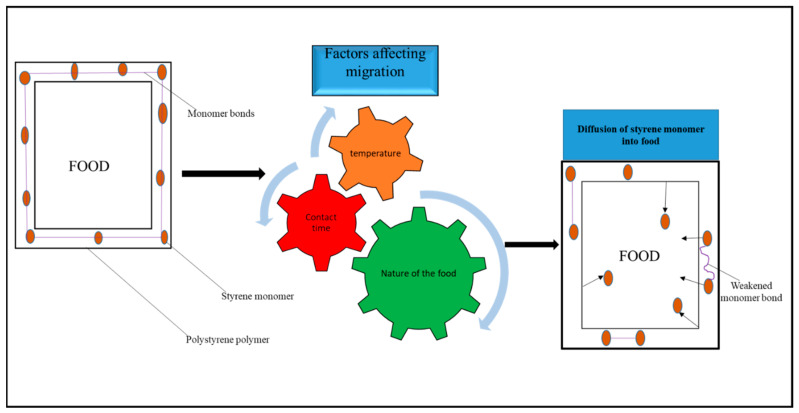
Migration mechanism of styrene from polystyrene.

Styrene monomer migration (Figure 9) is primarily a diffusion-controlled process that follows Fick’s law [32].

In the presence of elements influencing migration, the styrene monomer diffuses from a higher-concentration zone (the polystyrene food packaging) to a lower-concentration zone, which is the food, as result of weakened monomer/polymer interactions and increased solubility [126]. 

### 4.3. Factors Influencing the Migration of Food-Packaging Monomers into Food

Several factors govern the rate (kinetics) and general migration process from a food-packaging material into food [104,105]. The factors include aspects relating to the properties of the polymer material in interaction with the food (permeability, thickness, size, type, and format) and the properties of the migrant (polarity, molecular size, structure, and vapour pressure) as well as the state/properties and composition of the food materials, the starting migrant concentration in the packaging, the polymer matrix state, and the migrant components in contact with the food packaging [115,127]. The storage time, temperature, packaging size, period of contact, food surface area in relation to its volume such as with pasta, and packaging surface area ratio to the food product volume also affect chemical migration [105]. However, the primary factors affecting the migration process are as follows:

#### 4.3.1. Nature of Foods

The food simulants and different foods utilized so far to show the impact of the nature of food on packaging substance leaching depict that foods with excess fat have significant migration rates [128]. The Bisphenol A migration studies, for instance, during the storage and can denting of PC containers with carrots in brine (0% fat), evaporated milk (8% fat), minced beef in gravy (20% fat), and spring vegetable soup (0.3% fat), displayed significantly higher BPA migration into 10% ethanol in fatty foods than in other foods. The detailed BPA migration results were 47.2 ± 5.1 µg kg^−1^, 49.8 ± 10.9 µg kg^−1^, 53.8 ± 7.6 µg kg^−1^, and 45.7 ± 5.0 µg kg^−1^, respectively [129]. The styrene migration studies also showed a migration increase with the fat content [110]. The above information is attributed to the lipophilic nature of the chemicals contained in the packaging materials. In a separate investigation, higher styrene migration levels were recorded in ethanol-containing solutions than those recorded in isooctane solutions. However, styrene did not migrate in aqueous food solutions [130]. A similar behaviour was also observed and noted for Ɛ-caprolactam migration in the nylon 6 packaging. The samples analysed included poultry breasts, ham, turkey blanquettes, and bologna packages kept at 72–100 °C for 1–4 h. Ɛ-caprolactam migration exceeded the EU set limit of 15 mg kg^−1^ in 35% of the bologna sausage packaging, 33% of the turkey blanquette packaging, and 100% of both the pate and poultry breast packaging [117]. However, due to the numerous benefits of spices, food is usually cooked with a single spice or a mixture of spices. Seeds such as cumin, which are also utilized to produce spices, contain volatile oils. Perhaps it is important to further conduct experiments that show the contribution of spices in terms of influencing the leaching of monomers into food from packaging.

#### 4.3.2. Nature of Contact

Research studies indicate that there is a relationship between migration rates and the nature of contact (direct or indirect) between food and the respective contact material. The mass transfer of the chemicals from the packaging to the food increases when there is direct contact between the food and the packaging material. Compared to direct contact, in an indirect medium, the gas medium between the packaging and food causes slower migration [131].

#### 4.3.3. Period of Contact

Chemical migration largely depends on the duration of contact between the packaging and food [99]. Ref. [132], for instance, conducted research on the potential for PET oligomers to migrate from plastic packaging to different beverages and foods in ovens and microwaves at various temperatures with a focus on the temperature and exposure duration. Compared to oven heating, microwave heating showed less migration because of the shortened exposure time (maximum of 15 min for MW and 80 min for oven heating). Ref. [104] also highlights that, depending on the nature of the food, for instance, solid or liquid, oily or aqueous, and a moisture or fat content, the food-packaging material compatible at the beginning of the shelf life may become incompatible at the end of the shelf life. With time, for instance, foods that contain water are likely to draw polar immigrants, while fatty foods attract nonpolar immigrants. The conclusions based on the research studies therefore indicate that the square root of the contact time of the food and packing material determines how much of the mass of the migrant substance is transferred [133]. 

#### 4.3.4. Temperature during Contact

The temperature of the food directly influences the migration rate from the packaging into the food. In [128], it was discovered that migration rates rise as the temperature rises. Ref. [134] investigated styrene migration from various PS food-contact packaging materials, including egg cartons and meat trays, by exposing the materials to 8% ethanol and oil at 210 °C for 10 days, 490 °C for 4 days, and 65.5 °C for 1 day. The migration process exhibited a Fickian diffusion model. Migration increased from the first day to the tenth day and, for all materials with the exception of drink cups, was proportionate to the square root of the increase in time [112]. In a separate study on brand new PC baby bottles exposed to a temperature of 40–100 °C, the results showed a similar pattern, with the concentration of the BPA migrated into the food ranging from 0.03 ppb to 0.13 ppb at 40 °C to 95 °C, respectively [135]. Ref. [107] also used PC (baby bottles) and various other plastic containers (non-PC baby bottles) to study BPA migration into water and 10% and 50% ethanol. After 240 h at 40 °C, the average residual BPA content was higher in the 50% ethanol (2, 39 g L^−1^) than in the water (1.88 g L^−1^). The results showed that the higher the temperature and the longer the treatment periods are, the greater the BPA migration rate is [129]. Ref. [136] similarly came to the conclusion that the temperature has an inverse relationship with the log of the length of the equilibrium of a migratory material.

#### 4.3.5. Packaging Material Characteristics

The composition of a food-packaging material significantly impacts substance migration. The migration of monomer additives, for instance, is dependent on the packaging material’s thickness and plasticization. Thinner packaging allows for greater migration, and thicker packaging slows migration [21]. However, currently, research has not yet established any discernible relationship between the utilization of recycled components and the rate of migration [136].

#### 4.3.6. Migrant Characteristics

The nature of the migratory substance affects the rate and amount of migration. For instance, highly volatile materials migrate at a faster rate, and lower migration rates are shown for substances with significantly higher molecular weights [24]. However, some monomers, such as vinyl chloride and ethylene, migrate quickly even at ambient temperatures [28]. Migration is also affected differently depending on whether the migratory substance is spherical or branched. For instance, experimental findings demonstrate that branched molecules display slower migration rates [128]. Ref. [108] also explored the possibility of BADGE and BPA leaching into distilled water from two different can types: one used for jalapeno peppers and the other used for tuna fish. The findings showed an increase in migration with time during storage for the jalapeno pepper cans. Bisphenol A migration from the tuna cans was, however, independent of the storage time, while BADGE migration during storage decreased over time due to its instability and ability to hydrolyse in an aqueous medium. Overall, the BPA and BADGE migration levels ranged from 0.25 to 4.3 g kg^−1^ and from 0.6 to 83.4 g kg^−1^ [108].

#### 4.3.7. Migrant Concentration within the Packaging Material

Higher amounts of the migratory substances in the food matrix after a certain period of time in storage suggest that a mass transfer from the packaging into the food occurred at a higher rate as a result of a higher migratory compound concentration in the packaging material [137]. A study, for instance, conducted to investigate BPA migration under different factors, firstly involved processing the PC cans at 121 °C for 90 min and then storing them at 5 °C and 20 °C. Longer periods of storage were simulated by storing cans for up to 10 days–3 months. From the overall can coating BPA amount, the results showed 80% to 100% migration during processing. No BPA migration was observed in the simulants after processing. Therefore, the results suggested that there was a high migratory substance (BPA) concentration in the packaging material before processing [129].

#### 4.3.8. State of Polymer Matrix

This phenomenon refers to whether the polymer matrix exists at the storage temperature in a rubbery or glassy form. Migration in glassy polymers, such as PE, is substantially slower compared to that in rubbery polymers [32]. Due to the fact that migration can be reduced through migration-informed manufacturing or the use of specially developed low-migration closures, toxicological risk assessments of migrants are therefore utilized to set the migration limits for food-packaging materials. The limitations are incorporated into Food-Contact Regulations with the intention of limiting exposure to safeguard human health. However, in most countries, ordinary consumers have no access, or they lack knowledge on such regulations and, therefore, remain vulnerable until there is a national crisis that leads to the discussion of the issue in the media. By such a time, it is likely that there could be fatalities too.

#### 4.3.9. Migration Kinetics

Numerous factors affect the rate and speed of migration from food-packaging materials to food [99,104]. These include the features of the food-contact material, such as the thickness and permeability; the migrant chemical properties, including the molecular size, vapor pressure, polarity, structure, packaging material migrant’s original concentration, temperature, and contact time; and, furthermore, the nature of the food interacting with the packaging material, that is, either real food or stimulants [99,104]. Generally, for instance, small molecules, such as residual monomers, due to lower boiling points, migrate at a faster rate compared to larger ones [104]. Migration also increases significantly with increased temperatures that are accompanied by shorter contact times [99,104]. Migration also decreases with a decrease in the migrant starting concentration and the food-packaging material thickness. To the knowledge of the researcher, there is, however, no research to date that has compared the migration rates between real foods and simulants.

## 5. Interactions between Monomers and Food Nutrients

Once the monomer has leached, it combines covalently with the nutrients (Table 9) and/or the non-nutritive ingredients in the food. 

The interactions are based on the functional groups of the main nutrients usually present in the food and the leached monomers. Additionally, the processing technologies, storage conditions, and duration also play a significant role [138]. Table 9 illustrates some possible interactions between the main nutrients present in the food and the monomers (bisphenol A and styrene). 

## 6. Human Health Risks Due to Monomer Presence in Food

The initial food-packaging material regulations generally presupposed that, besides carcinogens, low-level chemical exposures, including EDCs, contained in food-packaging materials lower than the toxicologically determined no-effect levels had minimal health dangers to consumers [32]. However, to date, evidence from animal toxicological studies involving selected wildlife and human populations have raised more health questions than have been answered [139] (Table 10), for instance, in plastic food packaging, due to numerous health effects. 

However, the likelihood that consumers may experience negative health effects from any chemical contained in food mainly depends on the chemical toxicology and the exposure (dosage) as a result of the consumption of contaminated food. As such, currently, the utilization of ‘acceptable limits’ for different known chemicals is used to reduce the effects on humans. However, acceptable limits cannot exist for ‘unknown’ chemicals that, unfortunately, might be endocrine-disruptive and might have related or different adverse human health effects. This implies that, until a chemical is characterized and until their toxicological profile is determined, humans therefore remain vulnerable to their effects. There are several human EDC exposure routes, including the taking in of contaminated water and food, contaminated air inhalation, and chemical absorption through the skin, which are measurable using biological samples including breastmilk [89]. However, the consumption of contaminated food is continuously singled out as the major source of human exposure to EDCs across all age groups [144]. Once in the body, there are numerous independent toxicity actions that EDCs, including monomers, possibly interfere with, and they can block or imitate oestrogenic hormones, triggering diverse signalling pathways which yield diverse and divergent biological responses [145]. Alternatively, they could bioaccumulate in an organism’s lipid compartments and create mixed contaminated ‘body loads’ [89]. Currently, however, there are no studies that have focused on the impact matrices of EDC mixtures on the human body’s health. Table 11 presents the human health effects of some EDCs contained in food-packaging materials.

The extent at which humans are exposed to EDCs varies between countries due to variations in regulations [7]. The EU Member State and European Union (EU) regulations, for instance, list about 8030 chemical substances utilized in various food-packaging types [153]. However, in the United States (US) alone, about 10,787 chemical compounds are included in food both indirectly and directly as food additives [154], with many being used under the idea that they are generally recognized as safe (GRAS), even though they have not been reported to the US Food and Drug Administration (FDA) and have possibly not been tested to ascertain public safety. As a result, there is no published information on their use and possible exposure effects [45]. Presently, although in Africa, South Africa is amongst the few countries that control food-packaging materials mostly through general safety requirements due to its membership to the CODEX Alimentarius Commission. The Commission, created by the Food and Agriculture Organization (FAO) together with the World Health Organization (WHO) in 1963, creates international guidelines, food standards, and related texts like the codes of practice under the Joint FAO/WHO Food Standards Programme. In the above context, South Africans are protected by, for instance, the Foodstuffs, Disinfectants, and Cosmetics Act (FDCA) 54 of 1972, last reviewed as of 2009, which regulates food-related issues, such as importation into South Africa, including food packaging [155]. Specific laws that impact food-packaging materials are limited and lacking. They include R879/2011, which only forbids, among other things, the selling of Bisphenol-A-containing polycarbonate baby feeding bottles, and yet the BPA exposure of consumers occurs daily through everyday basic products, like food packaging. In addition, R962/2012 establishes the general hygienic standards for the transport of food and food premises.

According to Section 7(2) of R962/2012 [155]:

“A container shall be clean and free from any toxic substance, ingredient or any other substance liable to contaminate or spoil the food in the container”.

Given that South Africa has, as other upper–middle-income countries have, growing industries which attract an ever increasing population, food-packaging material use is therefore high, and standard consumer utilization practices vary and, in some instances, may possibly not be aligned with the material design. General regulations, such as those above, imply that producers continue to voluntarily comply with the expected standards and, hence, that substances with unacceptable hazard characteristics, such as reproductive toxicity, carcinogenicity, mutagenicity, bioaccumulation, and persistence or endocrine disruption, continue to be common in commerce, including their utilization in food-contact materials. Furthermore, the lack of consumer awareness concerning the chemical compounds in food packaging, such as plastics, and their effects on health continues, which greatly risks the health of consumers. Consumers in emerging countries, including South Africans, are therefore left to look after themselves by self-regulating legislation. There is therefore a significant likelihood that most people are unknowingly subjected to numerous individual and mixtures of FCC-associated chronic diseases [11,39,104]. 

## 7. Conclusions

Food packaging fulfils a significant duty in protecting food and enhancing people’s standard of living, and, therefore, the utilization of plastic food packaging globally cannot be expected to decrease any time soon because plastic is a unique material with numerous benefits. However, the monomers contained within plastic food packaging are a significant source of food chemical contamination, with endocrine-disrupting effects that affect both current and future human generations and environmental health. Unfortunately, research trends indicate that most monomers’ potential harm remains unaccounted for by science. Additionally, food migration studies utilize mostly food simulants rather than food products. Moreover, there is the broad consumer use and misuse of plastic packaging coupled with the nonawareness of the health concerns associated with its incorrect use. The European regulation on food packaging also continues to be criticized for its lack of revision to keep abreast with new scientific developments. Therefore, the research suggests that the entire human population is exposed to harmful substances with known and unknown effects on health from plastic food packaging. To safeguard both the present and coming generations, the scientific community has more work to do. The identification and understanding of the chemistries of both known and unknown food-packaging chemicals are more than urgent. Furthermore, food migration research studies need to be developed and need to shift their focus to real foods rather than food simulants to avoid generalizing the migration of a group of foods that may seem similar because they belong to the same category of foods and yet differ by one or two chemicals, which has implications in the migration process. Especially in countries with emerging economies, chemical migration awareness and knowledge is crucial and urgent for the relevant legislative authorities for the formulation, development, implementation, enforcement, and review of policies that advocate for sustainable packaging. Awareness and knowledge amongst the general population promotes reflection, which encourages behavioural changes towards the healthy utilization of plastic food packaging and towards checking on the manufacturing compliance with the legislation and regulations on the type of polymer used for food-packaging materials. 

## Figures and Tables

**Table 1:** Applications, pros, and cons of various food-packaging materials.

Type of Packaging	Applications (Types of Foods)	Advantages	Disadvantages	References
Plastics	Fast foods Solid products, such as pasta, rice, biscuits, bread, and sugar Liquid products, such as concentrate juices, oils, and methylated spirits	- Inexpensive materials - Thermosealability and microwaveability functional advantages - Optical properties - Unlimited sizes and shapes - Recyclable - Light weight - Strong - Oil and chemical resistance - Excellent barrier properties against gas and water vapour - Thermal stability and electrical insulation properties - Easily reused	- Residual monomer and chemical migration - Variable permeability to gases and light	[27]

**Table 2:** Composition of a plastic food-packaging material utilized to package yoghurt.

Packaging Material	Synthetic Materials Present	Food-Contact Chemicals		References
		Intentionally Added Substances (IASs)	Nonintentionally Added Substances (NIASs)	
Plastic packaging material	Aluminium Coatings Adhesives Printing inks	Monomers Oligomers Additives Pigments Metals	Impurities By-products of reactions Breakdown products Recycling-product contaminants Starting-material impurities Unwanted side products	[59,60,61,62]

**Table 3:** Chemical structures of monomers commonly used in common plastic food-packaging types.

Plastic Type	Recycling Code and Symbol	Monomer Name	Monomer Structure	References
Polyethylene terephthalate (PET)	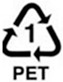	Ethylene terephthalate	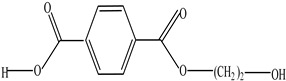	[65,66]
High-density polyethylene (HDPE)	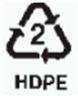	Ethylene	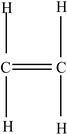	[65]
Polyvinyl chloride (PVC)	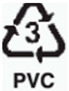	Vinyl chloride	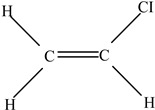	[65]
Low-density polyethylene (LDPE)	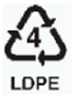	Ethylene	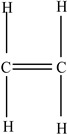	[67]
Polypropylene (PP)	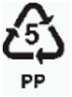	Propylene	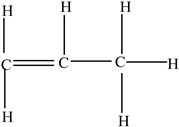	[65,67]
Polystyrene (PS)	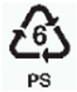	Styrene	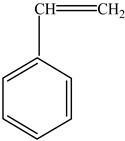	[65]
Other	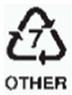	Bisphenol A for PC Caprolactam for Nylon-6 Bisphenol A diglycidyl ether for epoxy resins	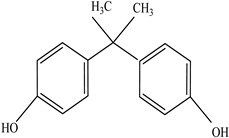 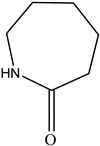 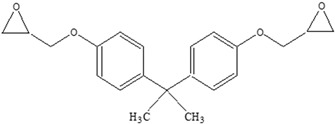	[68,69]

**Table 5:** Monomers for some common biodegradable packaging.

Biopolymer	Monomers	References
Polylactic acid (PLA)	Lactic acid	[74,76]
Polylactide aliphatic copolymer (CPLA)	Lactide + aliphatic polyesters	[74,76]
Polyglycolide (PGA)	Glycolic acid	[74]
Polybutylene succinate (PBS)	Glycols + aliphatic polyesters	[74]
PBAT	1,4 butanediol + terephthalic acid + adipic acid	[74]

**Table 6:** Uses of plastic food-packaging materials and EDCs contained in them.

Packaging Material	Structure	EDCs Identified in Such Compounds	Uses of Packaging	Ref.
Polypropylene (PP)	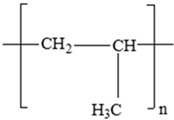	**Antioxidants** (vinyl and polymer with a methyl group) **Plasticizers** (phthalates)	Margarine tubs, microwaveable meal trays, lunch boxes, plastic bottle caps, and sweets and snack wrappers	[90,91,92]
Polyvinyl chloride (PVC)	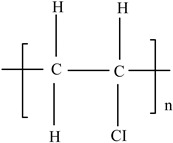	**Heat stabilizers** (Pb, Zn, and Sn compounds) **Dioxins** Plasticizers (phthalates) **Bisphenol A** (BPA)	Meat trays, bottles containing liquid foods (oils, vinegars, and beverage foods), flexible films for wrapping solid foods (fresh fruits, cheese, meat, and vegetables), coatings in metal cans, and lunch boxes	[93,94]
Polyethylene (HDPE and LDPE)	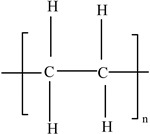	**Plasticizers** (phthalates) **Antioxidants** Ethylene and olefins (butene, hexene, and octene)	Freezer bags; milk cartons; yoghurt, fruit juice, and soup pots; caps for plastic bottles; Tupperware; plastic grocery bags; and shrink wrap	[25]
Polystyrene (PS)	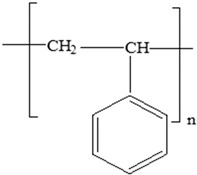	**Plasticizers** (phthalates) **Styrene**	Disposable coffee cups; plastic food boxes; containers for yoghurt, ice cream, fruit juice, and cheese; egg cartons; and biscuit trays	[24,25,51]
Polyethylene terephthalate (PET)	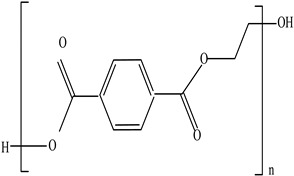	**BPA** **Phthalates** **Dioxins** **Colourants** **Fillers** **Plasticizers**	Water, soft drink, and alcohol beverage bottles as well as edible oil and fruit/vegetable punnets	[95,96]
Polycarbonate (PC)	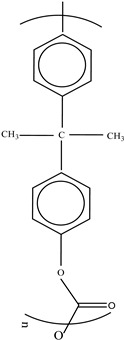	**BPA** **Phenol** **Volatile aromatic and aliphatic hydrocarbons** **Chlorinated hydrocarbons**	Recyclable beverage containers, ovenable frozen-food trays, and convenience meals	[97]
Polyamides (PAs)	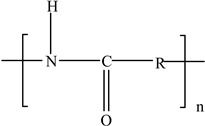	**BPA** **17α ethinyl estradiol** **Triclosan**	Vacuum packaging of frozen foods, bacon, cheese, and fresh and processed meats	[98]

**Table 7:** Residual styrene levels in PS packaging with similar products.

Country	Food Description	Residual Styrene Monomer Levels (µg/g)	Reference
Italy	Stirred yogurt, 3.2% fat	266 ± 1	[111]
Germany	Stirred yogurt, 3.5% fat	275 ± 2–351 ± 23	
Germany	Set yogurt, 3.5% fat	278 ± 12–308 ± 6	
Germany	Stirred sour cream with 10% fat	260 ± 8–292 ± 20	

**Table 8:** Bond dissociation energies for some plastic polymers utilized in food packaging.

	Different Methods’ Chemical Bond Average Values (kJ mol^−1^)								Ref.
Plastic type	Bond types								
	C-C bonds		C-CH_3_ bonds		C-C aromatic bonds		C–Cl bonds		
	M06-2X/6	B3P86/6- 31 G (d,p)	M062X/6 −31 G (d)	B3P86/6- 31 G (d,p)	M062X/6 31 G (d)	B3P86/6 −31 G (d,p)	M062X/6-31 G (d)	B3P86/6 −31 G (d,p)	
PE	364.3	350.9	-	-	-	-	-	-	[121,122]
	0.003	0.003							
PP	357.1	329.5	361.9	342.6	-	-	-	-	
	0.003	0.003	0.003	0.003					
PS	331.5	291.7	-	-	424.1	395.9	-	-	
	0.003	0.003			0.003	0.003			
PVC	373.8	345.8	-	-	-	-	355.6	343.7	
	0.003	0.003					0.003	0.003	

**Table 9:** Styrene and Bisphenol monomer interactions with functional groups of main nutrients.

Nutrients	Monomer	Reaction/Interaction
**Carbohydrates** 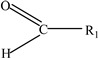	Styrene 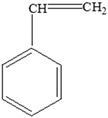	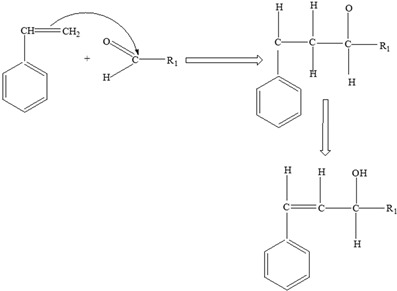
	Bisphenol A 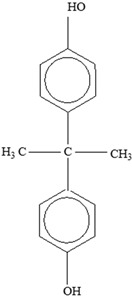	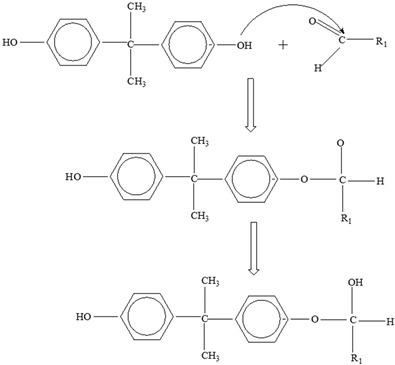
**Proteins** 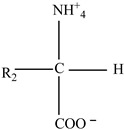	Styrene 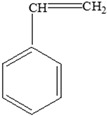	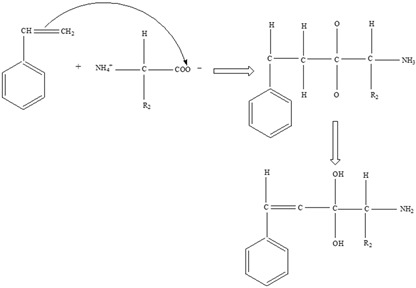
	Bisphenol A 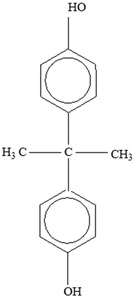	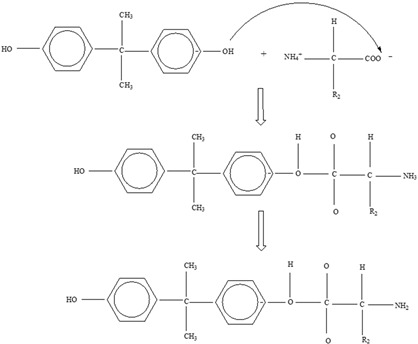
**Fats** 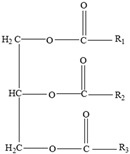	Styrene 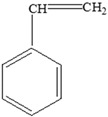	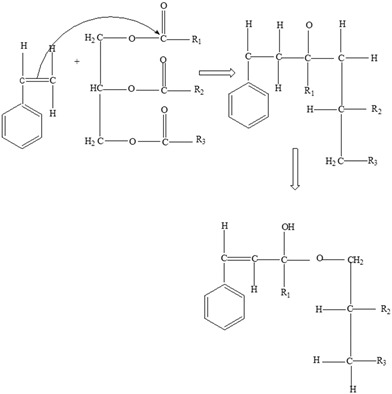
	Bisphenol A 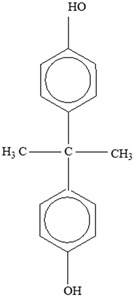	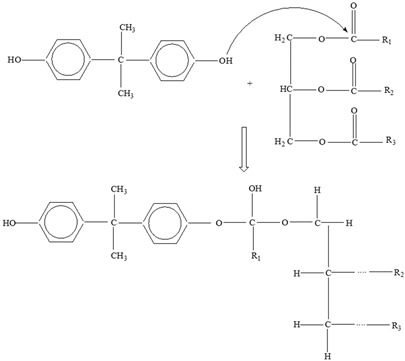

**Table 10:** Human health effects of some monomers contained in plastic food packaging.

Monomer	Health Effects	References
Styrene	-Toxic effect on the liver, chromosomal abnormalities, carcinogen, mucous membrane irritation, eye irritation, gastrointestinal effects, CNS dysfunction (reaction time and memory), effects on some kidney enzyme functions and on the blood, stimulates cell replication, cell proliferation, and cytogenetic damage promotion.	[36,140]
Vinyl chloride	-Liver, kidney, and lung toxicity; effects on liver, kidney, lung, spleen, nervous system and blood; cancer; causes steatohepatitis; affects glucose homeostasis; and enhances alcoholic liver disease.	[141]
Bisphenol A	Breast, ovarian, uterine, prostate, and testicular cancer.	[142]
Caprolactam	Cause neurasthenia syndrome and damages the central nervous system.	[143]

**Table 11:** Health effects of EDCs contained in food-packaging materials.

EDCs Present in Packaging Materials	Monomer Structures in the Food	EDC Health Effects	Sources
Plasticizers (phthalates)	**DMP** 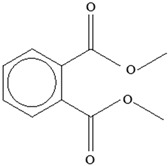 **BBP** 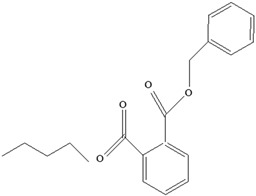 **DBP** 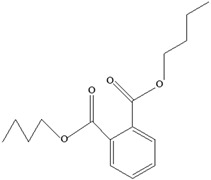 **DEP** 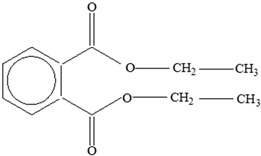 **DEHP** 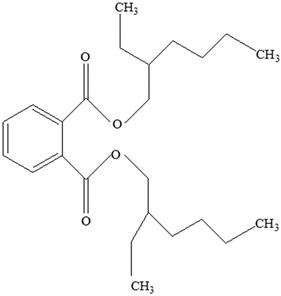	Has antiandrogenic effects when it interacts with the androgen receptor. Interacts with the aryl hydrocarbon (AhR) and PPAR receptors. Affects thyroid signalling. Reproductive disorders, including low sperm count. Reduced anogenital distance in males. Increased risk of preterm birth. Elevated oestrogen levels in pregnant women. Birth defects. Thyroid axis dysfunction in men. Asthma. Hypospadias. Cryptorchidism. Neurobehaviour problems.	[34,141,143,144]
Perfluoroalkyl substances (PFASs)	**Perfluoroalkyl carboxylic acids (PFCAs)** 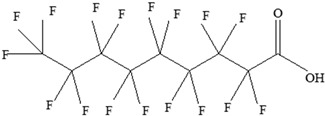 **Perfluoroalkyl sulfonic acids (PFSAs)** 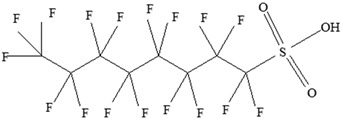	Decreased thyroid hormones. Has an effect on both pregnant women and children’s thyroid hormone levels. Increases hyperactivity. Developmental and immune toxicity. Cancer. Weight gain. Kidney and testicular cancer. Liver degeneration. Changes in nervous system development. Suppressed immune response. Decreased foetal and birth weights.	[145,146,147,148]
Dioxins	**Polychlorinated biphenyls (PCB)** 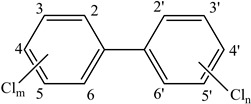	Increased metabolism. Suppressed concentrations of thyroxine. Reduction in blood insulin and glucose levels. Increase in serum gastrin. Infertility and foetal loss. Decreased spermatogenesis. Decreased circulating androgens. Endometriosis. Inhibition of growth factor and vitamin A expression. Ovarian dysfunction.	[149]
Styrene	**Styrene** 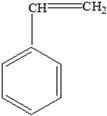	Reproductive toxicity. Developmental toxicity. Impaired immune response to concanavalin and reduced cell-mediated immunity. Neurotoxicity, which includes the suppression of the activity of the central nervous system, including slow reaction time and altered performance on neurobehavioural tests of memory and learning. Respiratory effects, including mucous membrane irritation. Gastrointestinal effects. Effects on the liver, kidney, and eye. Nasal irritation. Lung tumours.	[36]
Bisphenol A	**BPA** 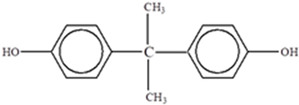	Oestrogenic properties. Interacts with a variety of nuclear receptors, including ERR, orphan receptor, oestrogen receptor, glucocorticoid receptor, human oestrogen-related receptor, PPARy, androgen receptor, and gamma receptor. Disrupts the thyroid axis. Causes metabolic disorders, which result in hyperactivity, neurodevelopment disorders, and type 2 diabetes. Causes infertility. Gut permeability. Breast and prostate cancers. It directly impairs oxidative homeostasis and indirectly impairs redox homeostasis by increasing oxidative mediators and reducing antioxidant enzymes. Increases hydrogen peroxide and lipid peroxidation. Alters organogenesis of kidneys, brain, and testes in foetus. Anxiety in childhood. Cardiovascular function disorders. Increases hydrogen peroxide and lipid peroxidation. In menopausal women, it can bind to ER (oestrogen receptor), triggering noxious cellular responses, such as binding to and stimulating oestrogen receptors (ERs) as well as disrupting action of other steroid hormones and DNA methylation. Disrupts normal action of androgens and alters thyroid hormone synthesis.	[150]
**Parabens**	**Methylparaben** 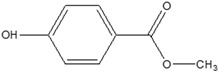 **Butylparaben, isobutylparaben** 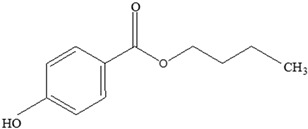	Exerts oestrogenic and antiandrogenic activities. Results in fecundity. Affects postnatal growth of boys. Increased weight. Cardiovascular diseases. More abnormal sperm. Lower testosterone levels. Cancer. Weakens enzyme activity that metabolizes endogenic hormones. Mimics oestrogens.	[35,151]
**Heavy metals**	**Cadmium** 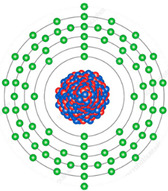	Cadmium, lead, mercury, and aluminium specifically linked to oestrogenic and breast-cancer-related effects. Mercury compounds also disrupt the thyroid gland function, the hypothalamic–pituitary–adrenal axis, and thyroid hormone function. Lead inhibits cellular enzymes and binding of sulfhydryl groups. It also affects membrane stability of red blood cells, inducing functional disturbances in peripheral nerves and development of the skeleton.	[152]

## Data Availability

No new data were created.
